# Fear of Death, Concept of a Good Death and Self-Compassion Among University Students in Portugal: A Cross-Sectional Study

**DOI:** 10.3390/healthcare13182382

**Published:** 2025-09-22

**Authors:** Marisa Pereira, Amira Mohammed Ali, Feten Fekih-Romdhane, Murat Yıldırım, Carlos Laranjeira

**Affiliations:** 1 School of Health Sciences, Polytechnic University of Leiria, Campus 2, Morro do Lena, Alto do Vieiro, Apartado 4137, 2411-901 Leiria, Portugal; marisa.pereira@ulso.min-saude.pt; 2 ULS Oeste–USF Rafael Bordalo Pinheiro, Rua do Centro de Saúde, s/n, 2500-941 Caldas da Rainha, Portugal; 3 Department of Psychiatric Nursing and Mental Health, Faculty of Nursing, Alexandria University, Smouha, Alexandria 21527, Egypt; amiramali@alexu.edu.eg; 4 Faculty of Medicine of Tunis, Tunis El Manar University, Tunis 1068, Tunisia; 5 The Tunisian Center of Early Intervention in Psychosis, Department of Psychiatry, Ibn Omrane, Razi Hospital, Tunis 2010, Tunisia; 6 Department of Psychology, Faculty of Science and Letters, Ağrı İbrahim Çeçen University, Ağrı 04100, Türkiye; 7 Psychology Research Center, Khazar University, Baku AZ1000, Azerbaijan; 8 Centre for Innovative Care and Health Technology (ciTechCare), Polytechnic University of Leiria, Campus 5, Rua das Olhalvas, 2414-016 Leiria, Portugal; 9 Comprehensive Health Research Centre (CHRC), University of Évora, 7000-801 Évora, Portugal

**Keywords:** fear of death, good death, self-compassion, survey, students, death education

## Abstract

**Background/Objectives:** Historically, humankind has consistently regarded death as an uncomfortable topic. Although death and dying are unescapable, they are frequently overlooked in formal education, as discussing or acknowledging them is believed to provoke emotional or psychological discomfort. To the best of our knowledge, little is known about the influence of the fear of death on the lives of university students. To fill this gap, this study aimed to examine the relationship between the concept of a good death, fear of death and self-compassion among university students in Portugal. **Methods:** This cross-sectional study was conducted in Portugal between November 2024 and January 2025 with 310 university students using an e-survey. Personal questionnaire and the Portuguese versions of the Good Death Concept Scale, the Collett-Lester Fear of Death Scale, and the Self-Compassion Scale were used. JAMOVI statistical software (version 2.7.6.) was used for descriptive analysis, independent sample *t*-tests, one-way ANOVA with post hoc analysis, and Pearson correlation analysis. To identify the factors associated with fear of death, a multiple linear regression analysis was conducted. This study adhered to the STROBE checklist for reporting. **Results:** A total of 310 students were included. The average age was 25 ± 8.52 years, and 75.2% were female. The total mean score for fear of death was 99.22 ± 21.97, indicating relatively low fear levels. However, health sciences students presented higher fear of death rates compared with non-health counterparts. Age and gender differences were also found, with female and younger students reporting significantly higher levels of fear of death (*p* < 0.01). The Pearson correlation matrix indicated that fear of death is positively correlated with the concept of a good death, while negatively correlated with self-compassion (*p* < 0.01). Key factors influencing fear of death include age, gender, closure and control domains, and the overidentification subscale (adjusted R-Squared valued [R2] = 0.352). **Conclusions:** The results suggest that students are often poorly prepared to deal with death-related issues (revealing fear) and with negative thoughts and feelings about mortality. In this vein, it is necessary to implement curricular educational interventions focusing on death education as well as actively involving students in compassionate community initiatives, increasing their awareness and self-confidence about EoL care.

## 1. Introduction

Although the perception of mortality has profoundly shifted in Western societies, the topic of death is still largely suppressed and absent from discussions about life [1]. Recently, advances in palliative care have fostered the acceptance of death and its integration into one’s life, helping individuals confront their fears of death. These developments have also promoted a growing interest within health and social sciences in a historically taboo topic [2,3].

Social scientists have also examined individual reactions upon recognizing the inevitability of death and dying. According to terror management theory (TMT) [4], mortality salience serves as a main catalyst for behavioral modification. When mortality is emphasized, responses range from denial of one’s susceptibility to death to feelings of sadness and anxiety [5], frequently resulting in a quest for a purpose in life. Death and dying present a challenge, namely the inability to regulate or foresee the timing and manner of their occurrence [4]. Anxiety and fear of death arise from the inevitability of mortality occurring at any moment. The fear of death can be intensified by news in the media, such as reports of terrorist attacks [6], mortality levels during the COVID-19 pandemic [7], the negative impacts of global climate change [8], or even a nuclear catastrophe resulting from the current Russia-Ukraine conflict.

The TMT posits that awareness of death’s inevitability (mortality salience) and the resulting fear of dying might generate a profound impetus to alleviate this anxiety (terror) through coping mechanisms (management) [9]. The theory highlights two mechanisms: cultural worldviews and self-esteem. Cultural worldviews are collective ideas that organize the universe by imparting a sense of order, meaning, and permanence. Greenberg et al. [4] assert that culture–by presenting the world as ordered, predictable, purposeful, and permanent–mitigates our existential anxiety by obscuring our fundamental creatureliness, which in turn encompasses our helplessness, vulnerability, and mortality. Self-esteem is the conviction that one conforms to cultural standards, cultivating a sense of one’s own worth. Self-esteem comprises two elements: (a) confidence in a specific cultural narrative that depicts human existence as substantial, lasting, and meaningful, and (b) the conviction that one occupies a vital position within that narrative. However, it has been considered somewhat inefficient in dealing with the purported psychological effects of the terror of death [10]. In this regard, self-compassion may be more important than self-esteem in managing death fear because it provides a more stable and resilient approach to dealing with negative emotions and existential concerns [11]. Unlike self-esteem, which can fluctuate based on perceived successes and failures, self-compassion offers a consistent sense of kindness and understanding towards oneself, even in the face of mortality [11]. Notwithstanding, together with cultural worldviews and self-esteem, self-compassion seems to be an antidote against the fear of death.

Self-compassionate individuals are marked by their openness to feeling pain and distress, by treating themselves with kindness, and by a self-awareness of their inadequacies [12]. They also understand that the negative feelings they experience are also experienced by others [13,14]. They confront these negativities and act compassionately towards themselves. Self-compassion is associated with adaptive psychological functioning, happiness, optimism, and other positive personality traits and positive psychological functioning, leading to wellbeing and psychological resilience [15,16,17,18,19,20]. Therefore, self-compassion can help people effectively confront their vulnerability as mortal organisms, a fate shared by all humanity, without resistance or complaint [21]. In turn, personal, familiar, social and economic distress often result in lower levels of self-compassion [22].

The Lancet Commission on the Value of Death highlights that how people die has changed radically in recent generations [23]. Death and dying have been relocated from the context of family and community to the domain of health systems. The role of families and communities has regressed, death and dying have become unfamiliar, and skills, traditions, and knowledge have been lost [23]. A good death is desired for all people and is a fundamental human right [24]. The concept of a “good death” refers to the notion that an individual can pass away comfortably and according to their preferences, whereby family members and medical personnel adhere to the dying person’s wishes [25]. Prior research indicates that an enhanced perception of a good death correlates with a more favorable attitude toward end-of-life (EoL) [26,27], avoiding “the ‘sequestration of death’—the process by which death is removed or distanced from everyday social life” [28]. However, the concept of a good death differs across cultural contexts. For instance, in the Global North, the focus is generally on the dying individual’s autonomy and wishes. Conversely, in the Global South, there is a heightened emphasis on the perspectives and requirements of family caregivers and social networks [28].

Although discussing the inevitability of death involves everyone, previous research has focused more on stakeholders’ perspectives on the dying process [29,30] and less on young, healthy adults. Furthermore, it is essential to empower generations of young adults to care for others, returning the dying process from the hospital setting to the community context. Likewise, death education among students fosters open discussions, reduces societal taboos, and equips individuals, including future healthcare providers, to deal with mortality-related issues [31]. Therefore, exploring students’ perceptions toward death is crucial to equip them with the skills, knowledge, and acceptance that will enable them to improve their psychological and spiritual adaptation to death.

To date, no Portuguese studies have directly examined the relationship between the concept of a good death, fear of death and self-compassion among university students. Our study aimed to: (1) assess the concept of a good death, fear of death, and self-compassion in higher education students; (2) determine the differences in students’ levels of death fear based on their personal characteristics; (3) assess the variation in the concept of a good death, fear of death, and self-compassion; (4) determine how variables related to the concept of a good death, fear of death, and self-compassion correlate; and (5) identify the factors associated with fear of death. We hope this study provides an overview of death-related issues in university students that contribute to structured learning experiences, addressing death as a natural part of life, reducing fear and anxiety surrounding mortality, and promoting a more positive outlook on EoL issues.

## 2. Materials and Methods

### 2.1. Study Design

A quantitative cross-sectional study was conducted according to the Strengthening the Reporting of Observational Studies in Epidemiology (STROBE) checklist [32].

### 2.2. Sample and Recruitment

The target population consisted of students from a public higher education institution in the central region of Portugal with a community of around 14,500 students. This institution offers undergraduate and postgraduate degrees in the areas of arts and design, marine science and technology, business and legal sciences, education and social sciences, engineering and technology, health and sport, and tourism. Eligibility criteria for participation were as follows: (a) individuals 18 years of age or older; (b) pursuing an undergraduate or postgraduate degree; (c) residing permanently in Portugal (>5 years); and (d) who understand Portuguese. Incoming international students were excluded due to language barriers. Moreover, students who were not accessible or available during the data collection period and not willing to participate were excluded.

### 2.3. Data Collection

Data were collected between November 2024 and January 2025 through self-completion of an e-survey using a snowball sampling approach through the institution’s mailing list and electronic communication tools (e.g., WhatsApp and Facebook), according to the principle of accessibility-availability. IP address filtering was used to lower the possibility of selection bias by preventing duplicate responses and responses from the same device. Completion of the instrument took, on average, about 10 min. The appropriate sample size was estimated using the G*power software version 3.1.9.7. [33] with a small effect size, a power of 0.90, an alpha at the 0.05 level of significance and a two-tailed test; the minimum required sample size was around 267 participants. At the end of data collection, 310 questionnaires were considered valid by the researchers (100% of the responses obtained).

### 2.4. Instruments

Data were collected through an online questionnaire (Google Forms) divided into two parts. The first addressed the participants’ personal information (age [years], gender [male; female and other], marital status [married/common law; divorced and single], and whether they had lost of a significant person in the last year [No/Yes]); academic information (field of study [health and non-health] and school year); and health variables (perception of physical, mental, and global health, measured on a 5-point Likert scale, with 1 being the worst possible state and 5 being the best possible state). The second part included three scales validated for the Portuguese population that assess the concept of a good death, fear of death, and self-compassion:

(1) The Good Death Concept Scale, developed by Schwartz et al. [34] and later translated and validated for the Portuguese population by Jordão and Leal [35]. This scale measures the relevance of several premises for each individual about what they consider a good death. The scale presents 11 items with Likert-type response options ranging from 1 “Not necessary” to 4 “Essential”, grouped into three domains: the Closure domain (reflects psychosocial aspects of a good death), the Control domain (focuses on the physical aspects of the death experience), and the Hope domain (related to the spiritual component of a good death). The total score varies from 11 to 44; a higher score on the scale indicates a greater perception of a good death. In this study, the Cronbach alpha coefficient of the scale was found to be 0.73.

(2) The Collett-Lester Fear of Death Scale (CL-FDS), developed by Lester and Abder-Khalek [36] and translated and validated for the Portuguese population by Pereira [37]. This multidimensional scale assesses fear of death and dying of self and others. It is composed of 28 items divided into four subscales (each containing seven items): Own Death, Own Dying, Death of Others, and Dying of Others. Responses are Likert-type and range from 1 (not at all) to 5 (very much). The total score ranges from a minimum of 28 points, indicating no fear of death, to a maximum of 140 points, reflecting the highest level of concern about death. In this study, the overall scale revealed excellent internal consistency with a Cronbach’s alpha of 0.94.

(3) The Self-Compassion Scale, developed by Neff [14], and translated and validated for the Portuguese population by Castilho et al. [38]. The scale consists of 26 items that assess the three basic components of self-compassion: Self-kindness (caring toward self), Humanity (or Common Humanity, i.e., understanding that all people make mistakes and struggle), and Mindfulness (awareness of the present). The items are grouped into six subscales: Self-kindness (5 items); Self-judgment (self-criticism) (5 items); Common Humanity (4 items); Isolation (4 items); Mindfulness (4 items), and Overidentification (fixation on negative thoughts/feelings) (4 items). Each item is scored on a 5-point Likert scale (1 = Almost never; 5 = Almost always). The total score is obtained by summing the scores of all items, where self-judgment, isolation, and overidentification subscales contribute negatively (reverse-scored items). Higher scores indicate higher levels of self-compassion. Applying Cronbach’s alpha coefficient, an excellent internal consistency was obtained in this study (α = 0.927).

### 2.5. Data Analysis

The frequency statistics (absolute and relative) of the qualitative variables and the descriptive statistics of the quantitative variables were analyzed using measures of location (mean and median), dispersion (standard deviation), and range (minimum and maximum). The first and third quartiles (the 25th and 75th quartiles) were analyzed, as well as the interquartile range (i.e., the difference between the third and first quartiles), to characterize the distribution and concentration of the data.

Prior to the study, data screening and cleaning were performed, and the assumptions of normality were assessed using the Kolmogorov–Smirnov test with Lilliefors correction (given *n* > 30) and linearity [39]. To compare the mean values of the scales between two independent groups, Student’s *t*-test for independent samples was used, considering the alternative hypothesis of statistically significant differences between the group means. To compare the mean values of the scales between three or more groups, one-way analysis of variance (ANOVA) was used. In groups where the assumption of homogeneity of variances between the compared groups was not met, Welch’s correction was applied. For variables where statistically significant differences between the means were found, post hoc multiple comparison tests were performed, namely the Tukey test (for homogeneous variances) and the Bonferroni test (for heterogeneous variances). We interpreted effect sizes based on the recommendations of Cohen (η2 > 0.01 indicates a small effect; η2 > 0.06 indicates a medium effect; η2 > 0.14 indicates a large effect) [40].

To assess the explanatory power of a set of associated factors for the dependent variable “fear of death” (global scale), a multiple linear regression analysis was conducted, integrating variables of sociodemographic, academic, general health, the concept of a good death, and self-compassion. The statistical significance of the regression coefficients was tested using Student’s *t*-test. The null hypothesis was rejected when *p* < α, indicating a statistically significant contribution of the predictor to the model. The assumptions for applying the regression analysis were ensured: the absence of multicollinearity between the independent variables (Tolerance greater than 0.1 and VIF less than 5) and the independence of the residuals (Durbin-Watson coefficient less than 3). The normal behavior of the model residuals, with zero mean and constant variance, was also ensured, as well as the linearity of the independent variables relative to the dependent variable [40].

Statistical analysis of the results was performed using JAMOVI software (version 2.7.6.). A significance level (α) of 5% (*p* < 0.05) was considered.

### 2.6. Ethical Considerations

The study received approval from the Ethics Committee of the Polytechnic University of Leiria (CE/IPLEIRIA/88/2024, approval date: 2 October 2024) and from the owners of the instruments. Ethical procedures followed the International Helsinki Guidelines [41], and students involved in the study were apprised of its content and requested to consent to voluntary participation, data sharing, and the privacy policy prior to their involvement. Participants completed the survey anonymously, directly connected to the Google platform. Participants could start the questionnaire only after providing informed consent by clicking “yes,” fully aware that they could withdraw from the study at any moment. There were no financial incentives for participation in the study.

## 3. Results

### 3.1. Sample Description

A total of 310 valid responses were obtained (Table 1). Participants ranged in age from 17 to 62; the average age was 25 ± 8.52 years. The majority (75.2%) identified as female and were single (81.9%). 35.5% of respondents reported having experienced at least one significant loss in the last year. Regarding academic characteristics, 48.1% of respondents were in the health field, with a greater representation of master’s degrees compared to other levels of study (28.1%). Global health status and physical health status were perceived with a midpoint of 3.5 [range 1 to 5]. Regarding mental health, the perception was slightly lower (3.1).

### 3.2. Good Death, Self-Compassion and Fear of Death Levels Among Students

The total mean score for good death was 35.76 ± 1.97. As presented in Table 2, the total mean scores for the good death subscales were as follows: Closure (9.67 ± 1.97); Control (17.34 ± 2.76); and Hope (8.75 ± 2.33). The total mean score for self-compassion was 75.22 ± 17.88. The total mean scores for the self-compassion subscales were as follows: Self-kindness (14.30 ± 4.32); Self-judgment (13.66 ± 4.42); Common Humanity (12.50 ± 3.46); Isolation (11.75 ± 3.78); Mindfulness (12.17 ± 3.34), and Overidentification (10.83 ± 3.45). The total mean score for fear of death was 99.22 ± 21.97. Lastly, the fear of death subscales had the following total mean scores: Own Death (21.38 ± 7.04), One Dying (25.90 ± 6.41), Death of Others (26.94 ± 6.25), and Dying of Others (25.01 ± 6.32).

### 3.3. Differences in Participants’ Level of Death Fear Based on Their Personal Characteristics

Statistically significant differences were observed in the mean fear of death global scores between females (M = 103.45; SD = 19.58) and males (M = 86.26; SD = 24.37) participants [t(97.27) = 5.403; *p* < 0.001] (Table 3). The mean difference was 17.19 points, with a 95% confidence interval between 10.88 and 23.51, and a moderate to large effect size (d = 0.778), indicating that women tend to report higher levels of fear of death than men.

Regarding age group, there were also statistically significant differences [F(2,306) = 5.229, *p* = 0.006]. Post hoc analysis indicated that participants aged 20–30 (M = 101.22; SD = 21.11) reported significantly higher levels of fear of death than individuals aged 31–40 (M = 91.94; SD = 23.20) and 41 or older (M = 89.68; SD = 25.14).

In terms of marital status, despite variations in means (single: M = 100.51; married/common law: M = 93.76; divorced: M = 91.60), the differences were not statistically significant [F(2,307) = 2.484, *p* = 0.085]. Regarding the experience of significant loss, the results indicated no statistically significant differences between those who experienced a loss (M = 100.06, SD = 20.70) and those who did not (M = 98.76, SD = 22.68) [t(308) = −0.499, *p* = 0.618].

Health students (M = 102.69, SD = 19.25) reported higher levels of fear of death than students in other fields (M = 96.02, SD = 23.83) [t(302.57) = −2.718, *p* = 0.007]. The mean difference was −6.67 points, with a small effect size (d = −0.308). No significant differences were observed regarding year level [F(7,302) = 1.425, *p* = 0.195], which means that the fear of death does not differ significantly depending on the year that students attend.

### 3.4. Variation in Study Variables According to the Students’ Field of Study

When applying the student’s test for independent samples (Table 4), there were significant differences in the subscales of the concept of Good Death and in the CL-FDS subscales (except for dying of others, *p* = 0.692), with higher values in the group of health students. Regarding the Self-compassion scale, no significant differences were observed between the two groups, *p* > 0.05.

### 3.5. Correlation Analysis Between Study Variables

Pearson’s correlation coefficient was used to assess the relationship between the different scales (Table 5). The results revealed that the overall Good Death Concept scale and its subscales present moderate (less than 0.50) to highly significant correlations with the overall CL-FDS scale and subscales.

Specific associations were observed between the Good Death Concept scale and the Self-Compassion scale. The Closure dimension presented a significant negative correlation with Isolation (r = −0.136, *p* < 0.05). The Hope dimension presented a highly significant positive correlation with Self-Kindness (r = 0.148, *p* < 0.01) and a significant positive correlation with Human Condition (r = 0.145, *p* < 0.05). In turn, the overall Good Death Concept scale showed a significant negative relationship with Overidentification (r = −0.119, *p* < 0.05).

The overall Self-Compassion scale and subscales had a negative and statistically significant effect on Fear of Death (overall scale and respective subscales). However, the correlations between Self-Kindness and “Own Dying”, as well as between common Humanity and “Own Death”, “Own Dying” and “Death of Others”, were not demonstrated statistically significantly.

All Fear of Death subscales showed strong positive correlations (above 0.70) and were highly significant with the respective overall instrument.

### 3.6. Influencing Factors of Fear of Death

Using the fear of death scores as the dependent variable, factors such as age, gender, marital status, field of studies, health perception, along with self-compassion subscales scores and concept good death subscales scores that are correlated with fear of death, were utilized as independent variables for multiple linear regression analysis. The results revealed that factors such as age, gender, closure, control, and overidentification scores are significant explanatory variables of fear of death among university students (*p*  <  0.05) (Table 6). The final model presented an explanatory power of 35% (adjusted R-Squared valued [R2] = 0.352). The overall F-test of the model was statistically significant [F(17.285) = 10.645, *p* < 0.001], confirming that the influencing factors, together, contribute significantly to explaining the variance in fear of death (global score).

## 4. Discussion

This study highlighted promising results on the fear of death, the concept of a good death, and self-compassion among university students. Deeply embedded in Catholic roots, Portuguese people give significant emphasis to values such as family, friendships, employment, and leisure. In addition, concepts like afterlife, salvation, and the significance of death and hope substantially influence individual views and coping strategies with mortality, alleviating anxiety and terror linked to death and dying [42]. Based on these assumptions, our results reveal that Portuguese people exhibit reduced global levels of death anxiety when compared to Eastern counterparts [43,44].

The data indicate that students fear the death of others more than their own death. The average scores on the CL-FD scale align with previous research concerning both the score range and the factors addressed by the four subscales, with the highest scores recorded for “Death of Others” and “Dying of Others,” and the lowest for “Own Death” and “Own Dying” [45,46,47,48]. Results suggest that students do not feel prepared for the death of another, which poses significant challenges for all those who may assume the role of formal or informal caregivers of people at the end of their lives.

The various dimensions of the Fear of Death scale (except for “Dying of Others”) obtained significantly higher values in students from the health field. These findings can be explained by their theoretical knowledge about death-related issues, as well as the personal experiences and academic education gained in clinical training in EoL care [49,50]. Being in the presence of a dying person can be an uncomfortable reality as it prompts us to reflect on our own existence and its end [51]. On the other hand, the romanticized idea that healthcare professionals have a mission to protect and prevent death persists, justifying the fear of others’ death.

Comparing with prior research with nursing students (112.2  ±  18.6) [47] and occupational therapy students (104.03  ±  16.576) [52], the total score of our study was 99.22  ±  21.97, signifying a lower level of fear of death. The disparities may be attributed to the demographic characteristics of the sample and the variations in the university education associated with the bachelor’s and master’s degrees. Our results are aligned with a previous study conducted with a French population, indicating that women exhibit a heightened fear of death compared to men, particularly in the subscales “Own Dying” and “Death of Others” [48]. Gender disparities were also noted among nursing students [53] in the subscales of the CL-FODS, specifically in the “Own Death” and “Death of Others” subscales, with females exhibiting a greater fear of death than males. Chuin and Choo [54] assert that men, like women, experience a fear of mortality but suppress or deny it. Furthermore, women typically serve as the principal caregivers for the dying, which may elucidate their heightened anxiety regarding death compared to their male counterparts [55,56]. Otherwise, age was shown to significantly correlate with death fear, indicating that as individuals mature, they acquire the resources needed to manage their fear of death more effectively. This outcome aligns with prior research [57], which indicates that the age disparity may stem from psychological maturity or growing spiritual awareness with age [48].

Based on the application of the Good Death Concept scale, and like the study by Jordão [58], the control domain presents the highest average, suggesting that the physical aspects of the process of dying are a main concern for a Good Death. The ability to manage dying activities is essential for accepting death, making choices, and preparing for the dying process. All domains of the Good Death Concept are significantly higher in healthcare students. Having to deal with the death of another in a professional context may translate into a greater appreciation of the premises that constitute the scale. Healthcare students frequently encounter EoL care throughout their academic experience and find it challenging and emotionally impactful. However, contact with death can constitute a threat if empathy and self-awareness are not exercised [59]. This scale showed a moderately significant relationship with the CL-FDS scale across all domains. That is, individuals with a higher Good Death Concept showed greater fear of death overall, suggesting that the motivation for greater appreciation of particular aspects of the dying process may be fear of death. Fear of death can paradoxically make us more driven and conscientious about how death can be good [60]. Students also presented lower overidentification scores on the self-compassion scale, showing less rumination and self-aversion reactions to negative events [61].

Although self-compassion levels were low in the sample, they were negatively correlated with fear of death. This may stem from the fact that individuals with elevated self-compassion are capable of treating themselves with greater kindness and assessing their fears about dying more openly [11]. Additionally, individuals with higher levels of isolation placed less value on the elements related to “Closure” in the Good Death Concept. This may make sense in light of the devaluation of components more focused on socialization (the opportunity to say goodbye and the person having lived through an event they consider important). Notably, individuals with higher “Self-Kindness” and “Human Condition” scores, and therefore more predisposed to be caring and tolerant of self-failure, placed greater value on the more spiritual component of the good concept of death (i.e., hope). Simultaneously, research indicates that those possessing increased levels of self-compassion do not evade accountability for unpleasant occurrences, and in doing so, they maintain a lack of negative self-perception [13,38,62,63]. People exhibiting elevated self-compassion endeavor to comprehend themselves rather than assign blame during challenging circumstances, thereby mitigating fear and anxiety [64,65]. Those possessing strong self-compassion recognize that vulnerabilities and suffering are not exclusive to them; rather, others also encounter analogous circumstances.

Lastly, our findings suggest that fear of death might be linked to a desire for closure and related to a perceived lack of control over the dying process. Moreover, rigid patterns of overidentification might exacerbate fear of death. While TMT posits that overidentification with a cultural worldview (e.g., religion, nationality) can reduce this fear by providing a sense of meaning and purpose [9], other researchers show that intense overidentification can exacerbate fear of death, potentially due to the perceived loss of self or the threat to one’s group identity [66].

### 4.1. Study Limitations

This study has several limitations. Firstly, the cross-sectional design does not allow for the establishment of causal relationships in the associations found, and the responses are conditioned by contextual or situational factors that were not analyzed. With longitudinal designs, the factors investigated can be better understood. Secondly, the snowball sampling technique applied at a single higher education institution does not reflect the sociodemographic and educational characteristics of the Portuguese university population, making it impossible to generalize the data and potentially generating reporting bias. Indeed, most participants were women, and more respondents had higher than average levels of education. Future studies should have a more equitable gender representation and a varied sample in terms of geographical locations and cultural backgrounds to enhance representativeness. Thirdly, the questionnaires used are self-administered, which may lead to bias associated with what is considered socially desirable.

Aside from the aforementioned limitations, the present study possesses several strengths, including (a) the utilization of widely accepted and standardized self-reported measures, (b) the initiative to investigate death-related issues within educational contexts, and (c) the provision of valuable insights to evaluate and assist the most vulnerable students. Furthermore, the study underscores the significance of self-compassion as a beneficial factor that may mitigate unpleasant emotions, such as the fear of death. While the causality between self-compassion and other variables remains unverified, it is essential to gather prospective data and account for protective factors in death education planning within higher education institutions.

### 4.2. Implications for Practice

Ensuring that the topic of death is addressed in the educational curriculum can help reduce fear of death, deal with individuals at the EoL and acquire bereavement support skills [67]. In this vein, it is necessary to invest in death education, promoting higher willingness to discuss EoL decisions and decreased death anxiety, death avoidance, and fear of death [67,68]. Enhancing the death literacy through death café conversations, narrative techniques (e.g., life review) [69], creative arts-based interventions (e.g., digital storytelling and photovoice) [70] or hospice visits are needed for improving comprehension of EoL care and facilitating a dignified death [42]. Such events and educational strategies may facilitate enhanced engagement of university students and staff with the Compassionate Communities concept [71], reducing societal taboos and preparing individuals to navigate mortality-related challenges [72,73].

Likewise, workshops and simulations of authentic EoL care scenarios can also enhance attitudes toward death, develop emotional intelligence skills and improve self-efficacy [74,75]. Skills like empathy, communication and self-reflection in safe environments are rarely emphasized in curricula [72,76,77]. However, these qualities, associated with emotional intelligence and growth mindset, are paramount to developing a professional identity, fostering growth on personal, social, and professional levels, and positioning individuals as active meaning-makers [72,78,79]. The inclusion of death education in higher education seems to promote a change in attitude in the face of death for students, helping them to see death as a natural process and developing a more compassionate attitude towards EoL patients and their families [80]. Exploring these scenarios is even more important as recent studies have failed to demonstrate a significant impact of psychosocial interventions on death fear and anxiety among adults [81].

Ultimately, this study emphasizes the importance of public health palliative care education initiatives (e.g., Last Aid courses [82]) in enhancing care for people with life-limiting illnesses and boosting community awareness about death-related issues [83]. Increasing access to these educational tools will enable more individuals to confidently contribute to the accessibility and effectiveness of palliative care in their communities [84].

## 5. Conclusions

This study contributes to the available evidence on fear of death among university students. It revealed how fear of death is related to students’ sociodemographic and academic characteristics, revealing particularly vulnerable groups: females, younger age groups, and those pursuing studies in the health field. It also clarified that young adults fear the death of others more than their own, which has important implications for future informal caregivers and health professionals in managing EoL processes. Fear of death is positively correlated with the concept of a good death, while negatively correlated with self-compassion. Key factors influencing fear of death include age, gender, closure and control subscales, and overidentification scores. Therefore, given the evidence found, doors of opportunity are opened for further studies, with a focus on developing interventions in the area of education/literacy about death and promoting self-compassion to create more compassionate educational communities rooted in a health promotion approach to palliative care.

## Figures and Tables

**Table 1:** Descriptive Characteristics of Students (*n* = 310).

Variables	Categories	*n* (%)
**Age (years)** Mean ± SD [range] 25.02 ± 8.52 [18–62]	20–30 years	251 (81.0)
	31–40 years	36 (12.0)
	41 or older	22 (7.0)
**Gender**	Female	233 (75.2)
	Male	70 (22.6)
	Other	7 (2.3)
**Marital Status**	Married/Common law	46 (14.8)
	Divorced	10 (3.2)
	Single	254 (81.9)
**Loss of a significant other in the last year.**	No	200 (645)
	Yes	110 (35.5)
**Field of study**	Health	149 (48.1)
	Non-Health	161 (51.9)
**Year Level**	1st year of the bachelor’s degree	59 (19.0)
	2nd year of the bachelor’s degree	53 (17.1)
	3rd year of the bachelor’s degree	54 (17.4)
	4th year of the bachelor’s degree	25 (8.1)
	Postgraduate studies	8 (2.6)
	Master’s degree	87 (28.1)
	Doctoral studies	2 (0.6)
		**Mean ± SD**
**Health Perception**	Physical	3.53 ± 0.81
	Mental	3.12 ± 0.97
	Global	3.51 ± 0.78

**Table 2:** Participants’ responses to scales under study (*n* = 310).

Variables	Mean	Median	SD	Min.	Max.	Percentiles		
						25	50	75
Closure	9.67	10.00	1.97	3	12	9.00	10.00	11.00
Control	17.34	18.00	2.76	5	20	16.00	18.00	20.00
Hope	8.75	9.00	2.33	3	12	7.00	9.00	11.00
Good Death (global)	35.76	36.00	5.27	14	44	33.00	36.00	40.00
Self-kindness	14.30	14.00	4.32	5	25	11.00	14.00	17.00
Self-judgment	13.66	14.00	4.42	5	25	10.75	14.00	17.00
Common Humanity	12.50	12.00	3.46	4	20	10.00	12.00	15.00
Isolation	11.75	12.00	3.78	4	20	9.00	12.00	14.00
Mindfulness	12.17	12.00	3.34	4	20	10.00	12.00	14.25
Overidentification	10.83	11.00	3.45	4	20	8.00	11.00	13.00
Self-Compassion (global)	75.22	75.50	17.88	34	121	63.00	75.50	86.25
Own Death	21.38	22.00	7.04	7	35	16.00	22.00	27.00
Own Dying	25.90	27.00	6.41	7	35	22.00	27.00	31.00
Death of Others	26.94	28.00	6.25	7	35	24.00	28.00	32.00
Dying of Others	25.01	26.00	6.32	7	35	21.00	26.00	30.00
Fear of Death (global)	99.22	102.00	21.97	28	140	85.00	102.00	115.25

SD: Standard Deviation.

**Table 3:** Differences in Participants’ Level of death fear based on their personal characteristics (*n* = 310).

			Fear of Death							
		N	Mean	SD	Statistics	*p*-Value	Differences	95% CI	**Cohen’s d**	**Post Hoc**
**Gender**	Female	233	103.45	19.58	t(97.27) = 5.403	**<0.001**	17.194	[10.88, 23.51]	0.778	-
	Male	70	86.26	24.37						
**Age group**	20–30	251	101.22	21.11	F(2,306) = 5.229	**0.006**	-	-	-	20–30 > 31–40 > 41
	31–40	36	91.94	23.20						
	41 or older	22	89.68	25.14						
**Marital Status**	Single	254	100.51	21.91	F(2,307) = 2.484	0.085	-	-	-	-
	Married/Common law	46	93.76	20.92						
	Divorced	10	91.60	25.17						
**Significant loss**	Yes	110	100.06	20.70	t(308) = −0.499	0.618	-	-	-	-
	No	200	98.76	22.68						
**Field of study**	Health	149	102.69	19.25	t(302.57) = −2.718	**0.007**	−6.666	[−11.49, −1.84]	−0.308	-
	Non-Health	161	96.02	23.83						
**Year level**	1st year	59	103.59	21.08	F(7,302) = 1.425	0.195	-	-	-	-
	2nd year	53	101.36	21.91						
	3rd year	54	98.94	24.07						
	4th year	25	104.84	16.67						
	Postgraduate studies	8	101.88	17.93						
	Master	87	94.26	22.19						
	Doctoral studies	2	102.50	34.65						

Bold means *p* < 0.05; SD = standard deviation; CI = confidence interval; Cohen’s d = effect size.

**Table 4:** Comparison of factor mean scores between health students and students from other areas.

Variables	Non-Health		Health		*t*	*p*-Value	95% CI
	M	SD	M	SD			
Closure	9.29	2.12	10.08	1.7	−3.66	**<0.001**	**[−1.22; −0.37]**
Control	17.04	3.01	17.66	2.44	−2.01	**0.046**	**[−1.24; −0.01]**
Hope	7.98	2.32	9.58	2.05	−6.43	**<0.001**	**[−2.09; −1.11]**
Good death (global)	34.3	5.48	37.33	4.55	−5.62	**<0.001**	**[−4.15; −1.89]**
Self-kindness	14.25	4.37	14.36	4.27	−0.22	0.827	[−1.08; 0.86]
Self-judgment	13.33	4.47	14.01	4.35	−1.36	0.174	[−1.67; 0.30]
Common Humanity	12.63	3.72	12.37	3.15	0.66	0.512	[−0.51; 1.03]
Isolation	11.43	3.91	12.1	3.62	−1.55	0.121	[−1.51; 0.18]
Mindfulness	12.32	3.46	12.01	3.2	0.8	0.424	[−0.44; 1.05]
Overidentification	10.68	3.56	10.99	3.33	−0.77	0.440	[−1.08; 0.47]
Self-compassion (global)	74.64	18.57	75.84	17.16	−0.59	0.556	[−5.20; 2.81]
Own Death	19.98	7.02	22.9	6.76	−3.73	**<0.001**	**[−4.47; −1.38]**
Own Dying	24.72	7.03	27.17	5.4	−3.45	**0.001**	**[−3.84; −1.05]**
Death of Others	26.18	6.79	27.76	5.51	−2.26	**0.025**	**[−2.96; −0.20]**
Dying of Others	25.14	6.84	24.86	5.72	0.4	0.692	[−1.12; 1.69]
Fear of Death (global)	96.02	23.83	102.68	19.25	−2.72	**0.007**	**[−11.49; −1.84]**

M—mean; SD—Standard Deviation; *t*—Student’s test for independent samples; CI—Confidence Interval; Bold means *p* < 0.05.

**Table 5:** Correlation matrix (Pearson correlation coefficient).

	1	2	3	4	5	6	7	8	9	10	11	12	13	14	15	16
1. Closure	1															
2. Control	0.399 **	1														
3. Hope	0.386 **	0.231 **	1													
4. Good Death (global)	0.753 **	0.775 **	0.707 **	1												
5. Self-kindness	−0.021	0.019	0.148 **	0.068	1											
6. Self-judgment	−0.094	−0.071	0.024	−0.062	0.571 **	1										
7. Common Humanity	0.052	0.035	0.143 *	0.101	0.691 **	0.382 **	1									
8. Isolation	−0.136 *	−0.057	−0.007	−0.084	0.465 **	0.652 **	0.308 **	1								
9. Mindfulness	−0.079	−0.076	0.03	−0.056	0.690 **	0.465 **	0.635 **	0.458 **	1							
10. Overidentification	−0.120 *	−0.094	−0.058	−0.119 *	0.465 **	0.694 **	0.357 **	0.687 **	0.524 **	1						
11. Self-compassion (global)	−0.085	−0.051	0.062	−0.031	0.833 **	0.817 **	0.707 **	0.762 **	0.789 **	0.789 **	1					
12. Own Death	0.417 **	0.259 **	0.244 **	0.399 **	−0.114 *	−0.175 **	−0.065	−0.199 **	−0.248 **	−0.264 **	−0.222 **	1				
13. Own Dying	0.386 **	0.312 **	0.318 **	0.448 **	−0.091	−0.242 **	−0.042	−0.242 **	−0.208 **	−0.325 **	−0.242 **	0.719 **	1			
14. Death of Others	0.335 **	0.244 **	0.237 **	0.358 **	−0.149 **	−0.267**	−0.069	−0.283 **	−0.243 **	−0.349 **	−0.288 **	0.570 **	0.615 **	1		
15. Dying of Others	0.295 **	0.288 **	0.118 *	0.313 **	−0.189 **	−0.320 **	−0.132 *	−0.317 **	−0.295 **	−0.380 **	−0.345 **	0.534 **	0.570 **	0.701 **	1	
16. Fear of Death (global)	0.426 **	0.326 **	0.272 **	0.451 **	−0.160 **	−0.294 **	−0.091 *	−0.306 **	−0.294 **	−0.388 **	−0.323 **	0.846 **	0.860 **	0.848 **	0.824 **	1

* *p* < 0.05; ** *p* < 0.01.

**Table 6:** The Multiple Linear regression analysis of fear of death among students.

Explanatory Variables	B	SE	*β*	*t*	*p*
Constant	73.747	11.286	-	6.534	< 0.001
Age (years)	−0.363	0.179	0.120	−2.025	**0.044**
Gender (Ref. Female)					
Male	−7.577	2.774	3.521	−2.732	**0.007**
Marital status (Ref. Married/Common law)					
Divorced	1.895	6.593	3.621	0.287	0.774
Single	1.478	3.777	2.100	0.391	0.696
Field of study (Ref. Non-Health)					
Health	2.432	2.324	1.987	1.046	0.296
Physical Health status	−1.808	1.636	−1.236	−1.105	0.270
Mental Health status	−1.019	1.669	1.020	−0.610	0.542
Global health status	3.490	2.192	2.632	1.592	0.112
Closure	2.652	0.635	1.470	4.174	**<0.001**
Control	1.080	0.410	0.998	2.634	**0.009**
Hope	0.848	0.524	0.699	1.620	0.106
Self-kindness	0.447	0.402	0.560	1.112	0.267
Self-judgment	−0.113	0.370	0.266	−0.307	0.759
Common Humanity	0.430	0.443	0.337	0.971	0.332
Isolation	−0.445	0.411	−0.321	−1.084	0.279
Mindfulness	−0.916	0.488	−0.841	−1.878	0.061
Overidentification	1.330	0.487	1.379	2.732	**0.007**

B—unstandardized regression coefficient; SE—Standard Error; *β*—standardized regression coefficient; Bold means *p* < 0.05.

## Data Availability

All data generated or analyzed during this study are included in this article. This article is based on the first author’s Master’s dissertation in palliative care at the School of Health Sciences—Polytechnic University of Leiria (supervised by Carlos Laranjeira).
